# Analysis of the Structure of 14 Therapeutic Antibodies Using Circular Dichroism Spectroscopy

**DOI:** 10.1021/acs.analchem.4c01882

**Published:** 2024-09-10

**Authors:** Maria G. Bruque, Alison Rodger, Søren Vrønning Hoffmann, Nykola C. Jones, Jean Aucamp, Tim R. Dafforn, Owen R. T. Thomas

**Affiliations:** † School of Chemical Engineering, University of Birmingham, Edgbaston B15 2TT, U.K.; ‡ School of Biosciences, University of Birmingham, Edgbaston B15 2TT, U.K.; § Research School of Chemistry, The Australian National University, Canberra 2601, Australia; ∥ ISA,Department of Physics and Astronomy, Aarhus University, Aarhus 8000, Denmark; ⊥ Lonza Biologics, Slough SL1 4DX, U.K.

## Abstract

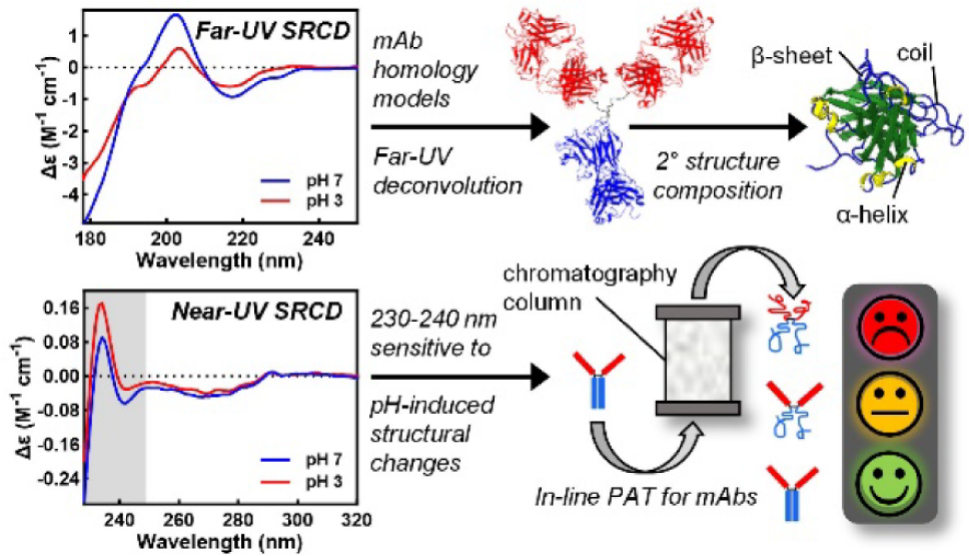

Understanding the impact of the manufacturing environment on therapeutic monoclonal antibody (mAb) structures requires new process analytical technology. Here, we describe the creation of a new reference set for the circular dichroism (CD) spectra of mAbs. Data sets of the highest quality were collected by synchrotron radiation CD for 14 different mAbs in both native and acid-stressed states. Deconvolution of far-UV spectra for the mAb cohort identified two current reference sets (SP175 and SMP180) as assigning accurate secondary structures, irrespective of the analysis program employed. Scrutiny of spectra revealed significant variation in the far-UV and especially near-UV CD of the 14 mAbs. Two spectral features were found to be sensitive to changes in solution pH, i.e., the far-UV positive peak at 201–202 nm and the near-UV negative exciton couplet around 230–240 nm. The latter feature offers attractive possibilities for in-line CD-based monitoring of the mAb structure during manufacture.

Since the approval of the first biotherapeutic protein, a recombinant insulin developed and manufactured by Genentech in 1982, the market for biopharmaceuticals has witnessed significant sustained growth.^1^ This growth is due in large part to the generation of monoclonal antibody (mAb)-based drugs,^2,3,4^ a hugely important grouping affording high specificities and low off-target effects,^5^ with current and projected (2032) global market sizes of >200 billion USD and >600 billion USD, respectively.^6^

A major preoccupation within the biopharmaceutical sector is to ensure that biotherapeutic protein products are correctly folded into authentic active 3D structures. Even seemingly trivial changes to the process environment during manufacturing can result in loss of structural integrity, which, in turn, compromises therapeutic efficacy and drug safety.^7,8^ Many factors that influence critical quality attributes (CQAs) of biotherapeutics have led to risk-based measures that safeguard product consistency, quality, and purity by ensuring that the manufacturing process remains substantially the same over time.^9^ Important among these initiatives is the FDA’s guidance on process analytical technology (PAT) within the pharmaceutical sector.^10^ PAT, as an enabler for quality by design (QbD), is increasingly employed in the development and monitoring of bioprocesses, often by reconfiguring/repurposing established laboratory-based analytics.^11,20^ Pertinent examples include: the use of ion exchange-based PAT for in-line monitoring of charge variants at all stages during mAb manufacturing;^11^ PAT based on coupling mass spectrometry with protein A affinity separation to detect shifts in the product glycan profile;^12,13^ in-line spectroscopic tools such as multiwavelength UV and FTIR for the quantification of protein species in mixtures based on identifying spectral signatures;^14,15,16^ and PAT for assessing protein aggregation both in-line and off-line.^17,18−20^ The aforementioned PATs can detect changes in CQAs in-process. They cannot, however, provide useful data on the changes in mAb secondary and tertiary structures in real time. Improved understanding of the impact of processing environments on the structure of biotherapeutics will benefit from the development of rapid, sensitive, and robust PAT for in-process monitoring/determination of a given protein’s structural state at all manufacturing stages.^10^ Moreover, outputs from such PAT could be used to control (feed-forward and/or feed-back) the process, ensuring that key parameters are optimized to minimize changes in the structural conformation and product yield.^10,21^

The propensity to aggregate, often accompanied by structural changes and/or improper folding in-process, is a major issue that leads to reduced yields, changes in particle size, and the presence of aggregates and large particles.^17,20,22−24^ The deployment of improved PAT methods to probe the protein structure hierarchy is likely to provide significant benefits to the biopharmaceutical industry, in particular for monitoring mAb structures during low pH elution, viral inactivation, and neutralization, all known to induce stress and increased aggregation.^22,23,25−27^

Circular dichroism (CD) is a sensitive spectroscopic technique commonly employed to determine the secondary structure and folding properties of proteins.^28^ Standard laboratory CD instruments are intended for measuring single samples and are not suitable for rapid handling of large numbers of samples or for use as inline detectors. In 2019, we introduced two novel CD PATs: first, an automated high-throughput low-volume sample (40 μL) system for combining CD and intrinsic protein fluorescence spectroscopy to collect complementary information on protein secondary and tertiary structures;^29^ second, one of the first CD spectrometers that could take real-time in-line measurements of mAb protein structures during chromatographic processes.^30^ The common and core feature of both setups – an optically compliant quartz capillary flow cell of a small path length (mostly 1 mm, but as short as 0.22 mm) – allows accurate and reproducible measurements in the far- and near-UV regions of the CD spectrum.^30^

Future development of CD-based systems as QbD-compliant PATs to underpin the manufacturing of all mAbs and related therapeutics will require a rigorously established reference set of mAb CD spectra. Although many works, including ours,^29,30^ report CD of individual mAbs, only a handful feature CD data collected for more than one in an identical manner.^31,32,33^ Consequently, most studies are of limited value in understanding how antibody structures influence the intensity and shape of CD signals. While the common β-sheet-rich immunoglobulin scaffold is the major contributor to CD signals in the far-UV region in all antibody structures, antibodies also contain a significant number of loop structures between strands and at domain boundaries, which also coincide with key regions of variability in the protein sequence and structure (e.g., the variable loops of antigen-binding sites).

In this study, the first of its kind, we identify features of antibody CD data that provide information about protein structural integrity. Specifically, we employed synchrotron radiation circular dichroism (SRCD) to collect the far- and near-UV CD data sets of the highest quality for 14 different therapeutic mAbs in both native and acid-stressed states. The resulting far-UV spectra were deconvoluted using commonly available algorithms. The success of each algorithm in predicting the secondary structure was assessed, culminating in a standard approach for deconvolution of CD data from mAbs. These data provide a new reference set for laboratories studying mAb structures while showing, for the first time, how different mAbs have different CD signatures and highlighting the importance of the near-UV region around 230–240 nm in indicating secondary structure perturbation.

## Experimental Section

### Materials

14 different mAbs (Table 1) were provided by Lonza at a range of stock concentrations and in different buffers. Sodium dihydrogen orthophosphate dihydrate (≥99.0%) and anhydrous disodium hydrogen orthophosphate (≥99.5%) were purchased from Fisher Scientific (Loughborough, Leics, U.K.), and all other materials were obtained from Merck KGaA (Darmstadt, Germany). All buffer solutions were filtered through 0.22-μm Durapore hydrophilic PVDF membrane filters (Merck KGaA, Darmstadt, Germany) and were prepared using deionized water. Prior to use, mAb samples were dialyzed into 50 mM sodium phosphate buffers of pH 3, 5 (for mAb2 and mAb4), or 7 using D-Tube Dialyzer Midi MWCO 6–8 kDa (Merck KGaA, Darmstadt, Germany). Protein concentrations were calculated from SRCD absorbance measurements at 280 nm, the correct extinction coefficient for the analyzed mAb, and the appropriate cuvette path length. Analysis of mAb sizes was performed by dynamic light scattering (DLS) in a Dynapro Plate Reader III instrument (Wyatt Technology, Santa Barbara, CA, USA). Samples (diluted to 0.5 mg/mL) were measured at 25 °C in triplicate with ten acquisitions of 5 s duration.

**Table 1:** mAbs Used in This Study

Sample	Isotype	LC type	VL family	VH family
mAb1	IgG1	kappa	VK3	VH4
mAb2	IgG1	kappa	VK1	VH3
mAb3	IgG1	lambda	VL6	VH5
mAb4	IgG1	kappa	VK1	VH7
mAb5	IgG1	kappa	VK1	VH3
mAb6	IgG1	kappa	VK1	VH3
mAb7	IgG1	kappa	VK4	VH3
mAb8	IgG1	lambda	VL2	VH3
mAb9	IgG4	kappa	VK1	VH1
mAb10	IgG2	kappa	VK1	VH4
mAb11	IgG4	lambda	VL3	VH6
mAb12	IgG1	lambda	VL1	VH1
mAb13	IgG1	kappa	VK4	VH1
mAb14	IgG1	kappa	VK1	VH1

### Panel of mAbs

Eleven IgG1, two IgG4, and one IgG2 with different combinations of kappa and lambda light chains and variable light chain (VL), and variable heavy chain (VH) families were investigated in this work (Table 1). The sample cohort represents the structural diversity commonly observed in mAb therapeutics, and allowed comparisons of individual family-type contributions to CD spectra to be made. All mAb samples were obtained post-protein A chromatography, and were assessed as >99% pure (based on peptide identity).

### Synchrotron Radiation CD (SRCD)

Spectra were obtained at the AU-CD beamline of the ASTRID2 synchrotron radiation source at ISA, Aarhus University in Denmark. Before data collection, the correct operation of the spectrometer was confirmed through the measurement of a spectrum of a known concentration of (1*S*)-(+)-10-camphorsulfonic acid (CSA). Far-UV measurements were collected from 280 to 170 nm in a cylindrical quartz cell (Hellma GmbH PC3& Co. KG, Müllheim, Germany) with a cell path length of 0.1015 mm at a protein concentration of approximately 1 mg/mL. Extending the upper wavelength to 280 nm permitted the precise determination of protein concentrations (see above) and baseline correctness. Near-UV measurements were recorded in a rectangular quartz cuvette (Hellma GmbH PC4& Co. KG, Müllheim, Germany) with a cell path length of 5 mm at a protein concentration of approximately 0.25 mg/mL. Spectra were collected between 330 and 230 nm to properly capture unusual spectral features in the frequently overlooked far–near UV transition region (230–250 nm). All spectra were collected in 50 mM sodium phosphate buffers of pH 7 (or pH 5 for mAbs 2 and 4) and pH 3. Sample and baseline measurements were collected at 25 °C using six and three scans, respectively, with a 1 nm wavelength step and 2 s average time per reading. To ensure accurate data collection, the low wavelength cutoffs of the CD spectra were chosen according to the overall absorbance of the system, defined by the high-tension (HT) voltage applied to the detector. All six sample scans were averaged, baseline subtracted, and smoothed with a 7-pt Savitzky–Golay filter.

### Far-UV CD Deconvolution

The secondary structure content of the panel of mAbs was estimated by deconvoluting the far-UV SRCD spectra on the online servers, DichroWeb^34^ and BeStSel.^35^ All samples were corrected for the concentration, mean residue weight, and cell path length to obtain molar amino acid residue CD data (Δε). The lowest SRCD wavelength used for analysis was 178 nm. Deconvolution was performed using the three most widely used analysis programs, CONTINLL^36−38^ SELCON3,^38^ and CDSSTR,^39^ with eight reference sets available on DichroWeb (sets 1, 3, 4, 6 and 7,^40,41^ SP175^42^ and SMP180,^43^ and also the BeStSel^35^ analysis program, which affords improved β-structure determination. The secondary structure assignments used were: “Helix” comprising regular α-helix (helix 1, αR) and distorted α-helix (helix 2, αD); “Sheet” consisting of regular β-sheet (sheet 1, βR), distorted β-sheet (sheet 2, βD), left- and right-hand-twisted antiparallel β-strand, relaxed antiparallel β-strand, and parallel β-strand for BeStSel; “Turns”; “Unordered” structures. To ensure consistency in secondary structure assignments, sets 2 and 5 were removed from the main analysis as they have different secondary structure classifications and provide additional helix content (3_10_-helix and polyproline II helix). The results from these reference sets are included in Tables S1–S3.

Differences between experimental and reconstructed CD data from the analysis program were assessed by comparing values of the goodness-of-fit parameter “normalized-root-mean-square-deviation (NRMSD)”:
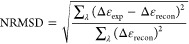
1where Δ*ε*_exp_ is the experimental molar amino acid residue CD, Δ*ε*_recon_ is the reconstructed molar amino acid residue CD, and *∑*_λ_ is the summation over all wavelengths.

The performance of the analysis programs was characterized using the root-mean-square-deviation, δ:
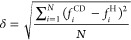
2where  is the fraction of secondary structure calculated from the deconvoluted CD data,  is the fraction of secondary structure calculated from antibody homology models, *N* is the number of samples, and  is the summation over all samples.

### Antibody Homology Models

Homology models were built in Molecular Operating Environment (MOE).^44^ Full-length light and heavy chain sequences were submitted to the antibody modeler application with “Ig” as the model type and refinement set to “1”. Selection of suitable framework templates for VL, VH, and CDR loops was done automatically, and fragment crystallizable (Fc) region templates were chosen from the MOE antibody library based on mAb isotypes: 1HZH^45^ for IgG1; 4HAF^46^ for IgG2; 5DK3^47^ for IgG4. Final models were optimized by applying a global energy minimization with a gradient of 0.1 RMS (root-mean-square). Additional parameters are given in Text S1 and Tables S4 and S5. The DSSP algorithm was used to assign secondary structure fractions to the final homology models using the online server DSSP-web^48^ as follows: α-helix (H) and 310-helix (G) structures were grouped as “Helix”; extended strand participating in β-ladder (E) was classified as “Sheet”; hydrogen-bonded turn (T) was assigned to “Turns”; residues in isolated β-bridge (B), π-helix (I), bend (S), and coil structures were grouped as “Unordered”. These secondary structure fractions were used as a reference for the performance of the deconvolution analysis.

## Results and Discussion

### Measurement of Far-UV CD Spectra of the Biotherapeutic Sample Set in Their Native State Using SRCD

**Figure 1:**
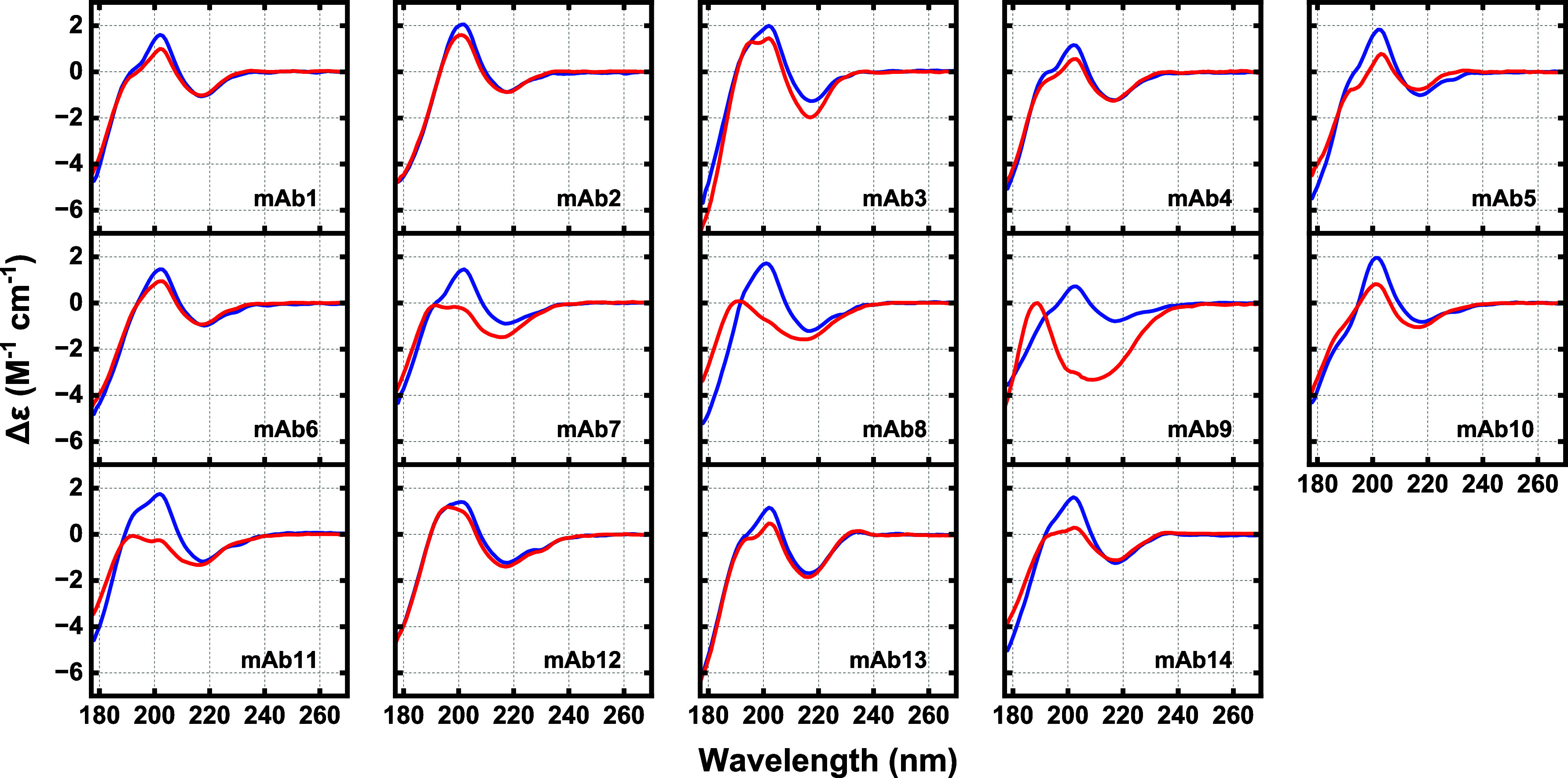
Far-UV SRCD for the 14 mAbs (Table 1) in 50 mM sodium phosphate at pH 5 or 7 (blue curves; pH 5 for mAbs 2 and 4, pH 7 for all other mAbs) and pH 3 (red curves).

In SRCD, the high flux of a synchrotron used as the light source enables the collection of high signal-to-noise CD spectra of proteins in milieus containing low molecular weight UV-absorbing species (e.g., buffers and salts) down to wavelengths close to the absorbance wavelength cutoff of water (∼176 nm with a 0.1 mm path length, or lower with shorter pathlengths). This data quality is important for a reference set, as discussed in Text S2. Accordingly, SRCD was employed to collect high-quality low wavelength data for all therapeutic mAbs listed in Table 1, in both native and acid-stressed states. The expectation of very similar/identical CD spectra for different mAbs, is understandable and widely held.^49^ However, the far-UV SRCD spectra for the 14 mAbs examined in their native states, i.e., at pH values of 5 or 7 (Figures 1 – blue traces and S1A), show quite significant differences. The general structure of the CD spectra at pH 7 is a negative peak at ∼217 nm, a positive peak around 201–202 nm, and a shoulder/peak at approximately 191–194 nm. The characteristic 217 nm negative peak, which is indicative of β-sheet structure, shows significant variation in its magnitude (e.g., ranging from −1.68 M^−1^ cm^−1^ for mAb13 to −0.79 M^−1^ cm^−1^ for mAb9). Such large differences in this prime region are somewhat surprising given the common Ig scaffold shared by all mAbs and may indicate that changes in the β-sheet content of the less structured regions of the mAb architecture influence the spectra. Below 200 nm, the changes in the spectral shape are even more significant. These include the presence or absence of a shoulder peak at 190 nm, and when present, this peak varies in intensity and position. Commonly reported far-UV CD spectra for antibodies miss this spectral feature.^26,31,33^ In spectra from a few mAbs (mAbs 8, 11 and 14) the shoulder around 190 nm is prominent and positive, whereas a greater number (mAbs 4–7, 9, 10, and 13) display lower intensity CD signals for this feature. In mAb3 and mAb12 spectra, the ∼190 and ∼200 nm bands merge strongly to form a broad peak not observed in the spectra for the other mAbs. Adverse effects on the quality and interpretation of CD data, i.e., baseline distortion at low wavelengths and increased light scattering, can frequently arise from the presence of unfolded molecules, β-sheet amyloid structures, high molecular weight aggregates, and suspended particles.^50^ To ensure that the presence of such entities did not interfere with SRCD data collection, all absorbance and HT traces were carefully analyzed (Figures S2 and S3). Good baseline matches to the appropriate buffer control readings were obtained in all cases, confirming the absence of aggregate interference and scattering in absorbance. These findings were consistent with DLS measurements of the mAb cohort in native vs. acid-stressed states (Figure S4). All samples were monomodal and monodisperse, and only small differences in the cohort’s average radius (6.2 vs. 6.9 nm) and polydispersity index (0.078 ± 0.038 vs. 0.098 ± 0.044) were observed, regardless of pH.

### Approach for Assessing the Effectiveness of Deconvolution of Far-UV CD Spectra of the Biotherapeutic Sample Set

Challenges to the deconvolution of CD spectra of antibodies are addressed in Text S3. An ideal approach for testing the accuracy of deconvolution would be to analyze CD data of proteins, for which a high-resolution structure exists. Unfortunately, high-resolution structures for therapeutic antibodies are scarce (either they do not exist or they have not been deposited in public databases). We did not have access to complete structures for any of the antibodies used in this work. However, crystal structures for CDR loops and framework regions of all 14 mAbs are known. Using the available crystal structure templates and the highly conserved structure of antibodies, we constructed homology models for all mAbs and employed these as surrogates for crystal structure references of the secondary structure contents of each mAb. A two-stage approach was used to assess the ability of the deconvolution algorithms to predict the mAb structure. In the first stage, CD spectra of all mAbs were deconvoluted using: (i) the algorithms CONTINLL,^36,37^ SELCON3,^38^ and CDSSTR,^39^ together with all reference sets available on DichroWeb;^34^ (ii) the BeStSel^35^ algorithm. These were assessed by examining NRMSD values (eq 1) for the deconvolutions. The NRMSDs compare calculated and experimentally derived spectra for each mAb. In the second stage, the performance and accuracy of algorithm predictions was evaluated by comparing the deconvolution results of all mAbs against the estimated secondary structure of antibody homology models using δ values (eq 2).

### Assessment of the Quality of the Deconvolution Analysis

The quality of the deconvolution analysis can be interpreted from values of NRMSD. Generally, low NRMSD values (<0.1) are taken to indicate a good quality deconvolution analysis.^51^ The spectral goodness-of-fit (NRMSD) used in this work, however, does not always correlate with structural goodness-of-fit (as estimated by δ from the homology model). NRMSD values obtained for mAb samples with all algorithms and reference sets are shown in Table S6. A “green-amber-red” color scale was employed, with green for low values of δ (good agreement between CD-derived and expected secondary structures) and red for poor agreement.

A general observation is that low NRMSD values (green) were obtained by the CDSSTR (av. 0.047) and especially BeStSel (av. 0.020) algorithms, while higher NRMSD values (amber-red) were recorded by CONTINLL (av. 0.168) and particularly SELCON3 (av. 0.393). CDSSTR provided suitable NRMSD values for all mAbs and reference sets, CONTINLL showed acceptable NRMSD values (<0.1) for a few mAb samples, but only when using SP175 (mAbs 2, 3, 5, 6, 8, 10, and 14) and SMP180 (mAbs 2, 3, and 14), and SELCON3 delivered unacceptable NRMSD values across the board, i.e., regardless of sample or reference set. The disparity in NRMSD values obtained by CDSSTR, CONTINLL, and SELCON3 algorithms derives from differences in the structural fitting of these three algorithms. It is widely accepted that a small CDSSTR NRMSD, though obligatory, is not necessarily sufficient for a good structure prediction. BeStSel provided the best deconvolution quality with the lowest NRMSD values, indicating a good fit between the experimental and reconstructed data. This finding was not unexpected. The BeStSel algorithm^35^ has been optimized to differentiate between β-sheet orientations and to provide higher accuracy and lower NRMSD for β-sheet-rich proteins (i.e., IgG and protein aggregates) cf. other deconvolution algorithms.^35,52^

It should be noted that high NRMSD values can result from incorrect measurements of concentration, path length, and/or unusual features in the CD spectra of the sample cf. that of the reference spectra. In this work, the concentration and path length were accurately determined prior to every CD measurement, so high NRMSD values correlate with a lack of relevant β-content reference set members for CDSSTR, CONTINLL, and SELCON3.^51^

The reference data sets employed in this study differ in (i) the wavelength range of the data used, (ii) the number, and (iii) type of proteins included. Analysis of the protein data employed reveals that four data sets (SMP180, SMP180t, SP175, and SP175t) include one immunoglobulin, specifically a full mouse IgG (1IGT).^53^ Data sets 1, 3, 4, 6, and 7 contain no immunoglobulin data, but all include data for an isolated lambda immunoglobulin light chain, i.e., the Bence Jones protein (2RHE).^54^ Overlaying the CD spectrum for the only IgG in data set SP175 (PCDDB ID: CD0000039100)^42,55^ on our experimentally obtained SRCD data for 14 therapeutic mAbs (Figure S5) highlights significant differences at around 190–200 nm (noted earlier), as well as at 230 nm except for mAbs 4 and 13. Thus, the IgG reference cannot accurately represent the diversity of spectral shapes in the far-UV region of mAbs; consequently, high NRMSD values are determined for most fits (Table S6; sets 1, 3, 4, 6, and 7). However, low NRMSD values alone are not sufficient to gauge the accuracy of results; these are best interpreted alongside the expected secondary structure content of the protein of interest.^35^

### Performance and Accuracy of Deconvolution Algorithms

Our homology models are consistent with DSSP of five full-length IgG crystal structures (see Table S7) available in the PDB (1HZH,^45^ 5DK3,^47^ 1IGT,^53^ 6GFE,^56^ and 1IGY^57^), so we are confident in using them as controls. Table S8 summarizes the performance of CONTINLL, SELCON3, and CDSSTR programs in determining mAb secondary structures in terms of δ. A “green-amber-red” color scale akin to that used for NRMSD (Table S6) is employed, with green for low values of δ (indicating good agreement between calculated and expected secondary structures) and red for the highest values (poor agreement).

It is evident that no one analysis program outperforms the others and that the reference set exerts the largest impact on δ. The accuracy of BeStSel, an algorithm designed for the analysis of proteins with high β-sheet content (such as the mAb samples in this study), was no greater than that of the other programs tested. The total helix content calculated by BeStSel was significantly underestimated cf. the expected value (δ = 0.07; Table S8), which may, in large part, reflect 3_10_-helix assignment to the “Unordered” (better referred to as “Other”) classification. Reference sets SP175, SP175t, SMP180, and SMP180t consistently gave more accurate secondary structure content, especially for the β-structure, regardless of the analysis program (CONTINLL, SELCON3, and CDSSTR) used. The best-performing data sets are those that feature an IgG; while this appears logical, it should be noted that this reference IgG (PCDDB ID: CD0000039100)^42,55^ exhibits quite different spectral features compared to the mAb cohort examined here (Figure 1). It is probable that data sets featuring the largest number of proteins, i.e., SP175 (71 proteins) and SMP180 (128 proteins), provide broader coverage of spectral features. All of the analysis programs tested here underestimated total helix contents by 2–5%. This is not unexpected as the helix contents of mAbs are very low. The best-performing combination of the analysis program and reference set for all mAbs tested at native pH (pH 5 for mAbs 2 and 4, pH 7 for all other mAbs), based on NRMSD (deconvolution quality) and δ (accuracy) values, was “CDSSTR + SP175”. This combination was therefore used to characterize the secondary structure content of all mAb samples at pH 3.

### Near-UV CD Spectra of the Therapeutic mAb Sample Set in Their Native State Using SRCD

**Figure 2:**
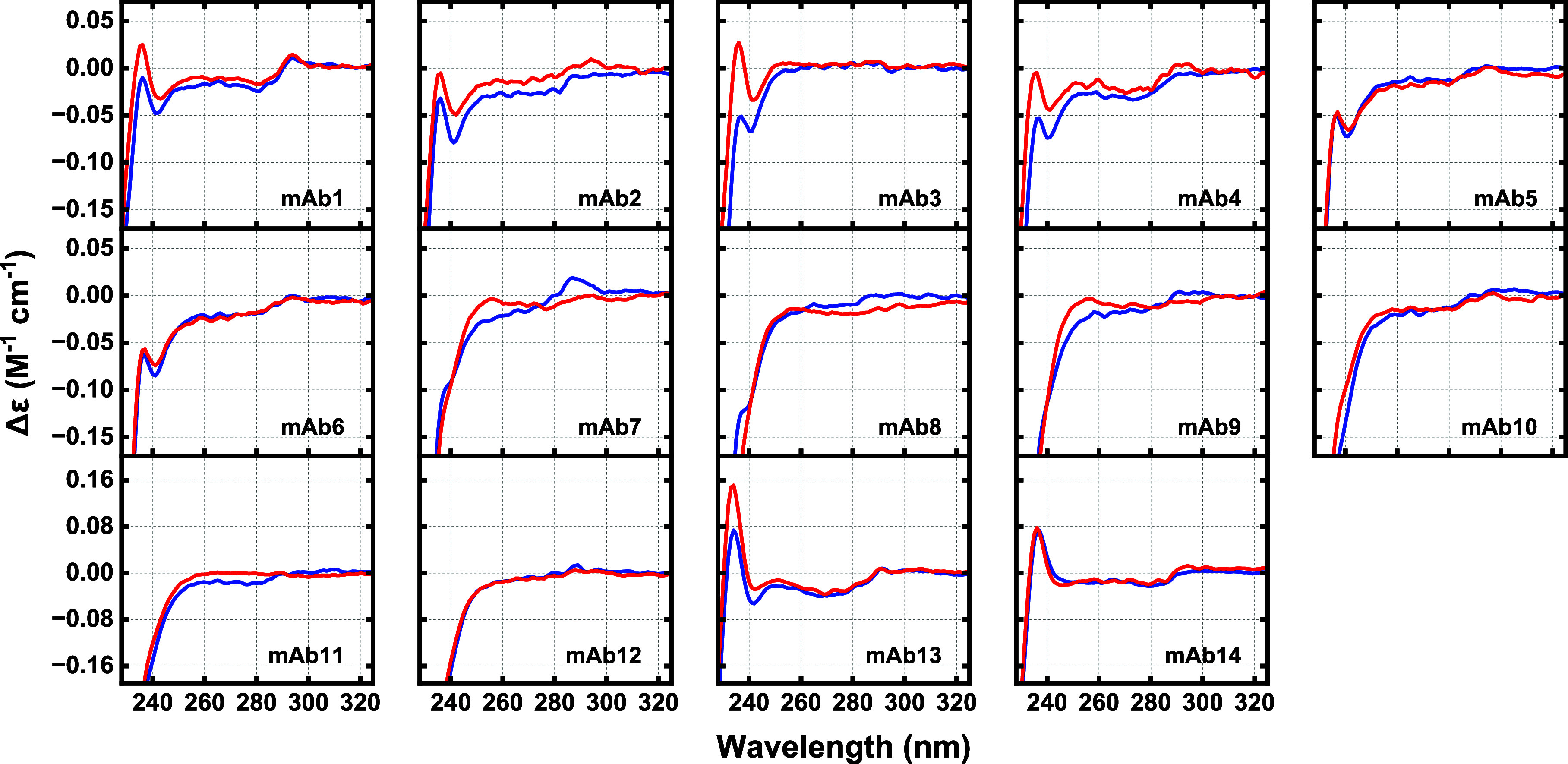
Near-UV SRCD for the 14 mAbs (Table 1) in 50 mM sodium phosphate at pH 7 (blue curves, pH 5 for mAbs 2 and 4) and pH 3 (red curves).

In common with observations presented of differences in far-UV SRCD spectra of the mAb cohort (Figure 1), clear variations are also discernible in near-UV SRCD spectra of mAbs (Figures 2 and Figure S1B). Variations in peak height at 290 nm (characteristic of Trp contributions) are evident, but the most prominent feature in the near-UV region at pH 5 or 7 is the presence or absence of a pair of opposed peaks occurring between ∼230 and ∼250 nm, i.e., a positive band at ∼230 nm and a negative band at ∼240 nm. This spectral feature is characteristic of a negative exciton couplet. Exciton couplets consist of two peaks of opposite sign and similar intensity, and arise when a chiral molecule, such as an IgG, contains two chromophores with electric-dipole-allowed electronic transitions in close proximity to and properly aligned with one another.^58,59^ The sign of an exciton couplet is described by the sign of the peak at a longer wavelength (in this case, negative at ∼240 nm) and depends on the absolute angle of twist of the dipole electronic transitions.^59,60^ The spectra for eight mAbs (mAbs 1–8) exhibit twinned peaks of similar size to one another but vary in exciton intensity. For example, mAbs 1–6 display high-intensity negative exciton couplets, whereas exciton intensity is minimal for mAbs 7 and 8. Twin peaks are wholly absent from the near-UV spectra of four antibodies (mAbs 9–12), and the 230–240 nm region shows strong negative intensity due to the tail of the backbone spectrum. Finally, the exciton couplet for the mAb13 spectrum comprises 230 and 240 nm peaks of unequal intensity (the positive peak at 230 nm being the much larger of the pair), whereas that for mAb14 shows a single positive peak at 230 nm but lacks an accompanying negative feature at 240 nm. The absence of twinned peaks and variations in peak intensity likely indicate that one or both chromophores involved in exciton coupling are missing and/or their conformation or spatial separation is unfavorable.

Contributions to the CD region between 220 and 250 nm are commonly attributed to the peptide backbone and the presence of disulfide bonds. Woody^61^ and coworkers emphasize that disulfide contributions in this region (and further into the near-UV) are typically broad and nonexcitonic,^62^ and importantly, aromatic side chains can also affect this region and, in some cases, are predominant.^61^ The variable region of antibodies is rich in Tyr and Trp residues, especially at the interdomain surface; it follows that aromatic contributions to near-UV spectra mainly arise from this region of the IgGs.^63^ Our observed couplets almost certainly arise from the coupling of two Trp or Trp–Tyr residues in the variable regions. Several lines of investigation support this theory. Using theoretical calculation, Woody^61,64^ and Manning^65^ showed that L_a_ transitions of Tyr and B_b_ transitions of Trp usually contribute to positive (though sometimes negative) spectral bands in the 225–230 nm region, whereas Phe contributes to wavelengths of <220 nm. Strong positive bands in the 225–250 nm region have been experimentally attributed to Tyr and Trp side chains for several proteins and theoretically attributed to immunoglobulins.^61,66^ CD spectra of Fab fragments bound to lysozyme^66^ and of Fab(t) fragments arising from tryptic digestion of normal and myeloma human IgG exhibit a negative band at around 241–243 nm and a positive band at around 232–235 nm. These bands are absent from the spectra of the corresponding Fc(t) fragments,^67^ which suggests a link between this characteristic twin peak spectral feature and the variable region of mAbs. The findings documented here (Figure 2) are the first to show the existence of a classical negative exciton couplet in certain therapeutic mAbs (mAbs 1–8; Table 1), and the absence (mAbs 9–12; Table 1) or permutation (mAbs 13 and 14; Table 1) of this feature in others. Exciton couplets are particularly sensitive to changes in pH, buffer composition, and temperature, given that these can influence the degree of exposure of buried aromatic chromophores to the solvent. From this, it follows that CD measurements in the 230–240 nm region can provide a convenient, and possibly generic, PAT for monitoring changes to a given mAb’s structure.^30^

### Detection of Protein Fold Disruption at Low pH Using CD

Key elements for the successful deployment of PAT in mAb bioprocessing are: (i) the ability to detect changes in structure that diverge from the ideal or “native” state; and (ii) that the generated signal changes in a predictable fashion should the mAb begin to denature or aggregate, clearly signaling the need for corrective action. Acid-induced unfolding (and aggregation) of therapeutic antibodies is of particular interest because protein A affinity chromatography, the main purification step used in almost all mAb downstream processes, relies on the use of a low-pH (typically pH 3–4) buffer for elution, and following elution, mAbs are held at low pH for long periods in the subsequent virus inactivation step.^22,30^ To assess the utility of CD in detecting deleterious structural changes induced by low pH, far-UV (Figure 1, red traces) and near-UV (Figure 2, red traces) SRCD spectra for all 14 mAbs were recorded at pH 3.

### Influence of Acid-Induced Protein Denaturation on Far-UV CD

Comparison of far-UV spectra recorded at near-native pH (pH 5 or 7) with those obtained at pH 3 emphasized that some antibodies (mAbs 1, 2, 6, and 12) experienced little apparent change in their secondary structure on exposure to acidic pH (spectra were only minimally altered), while others suffered significant structural disruption (mAbs 7–9 and 11) manifested by strong perturbation across most of the far-UV range and, in particular, by reductions in peak height at ∼200 nm. In extreme cases (e.g., mAb9), the decrease in the CD signal at 200 nm was accompanied by an increase at ∼214 nm, indicative of much larger conformational rearrangements in this mAb cf. some others (e.g., mAb1). These changes are analogous to those expected for a decrease in protein folding and correlate well with the transition to a spectral shape known to be associated with unfolded proteins.^33,68,69^ Secondary structure deconvolution using the CDSSTR algorithm and SP175 reference set is shown in Table 2 (NRMSD values of <0.1). Ten antibodies (mAbs 1–6, 10, 13, and 14) maintained native-like secondary structures at pH 3, whereas four (mAbs 7–9 and 11) experienced some degree of unfolding and loss of secondary structure as β-sheet content fell (by 5–9%) at the expense of combined increases in α-helix (up to 2–3%) and unordered structures (up to 1–5%). Previous studies for different antibodies have reported similar conversions of β-sheet to α-helix and random coil following exposure to low pH.^25,68,69^ These changes in CD, though by no means large, are significant, readily detected, and larger than the recorded differences between individual antibodies at near-native pH values of 5 or 7.

**Table 2:** Secondary Structure Fractions of the mAb Cohort at pH 7 and 3 Determined by the CDSSTR Analysis Program and the SP175 Reference Set

	pH 7					pH 3					Change in the secondary structure			
Sample	NRMSD	H	S	T	U	NRMSD	H	S	T	U	H	S	T	U
mAb1	0.041	0.02	0.51	0.09	0.36	0.037	0.02	0.50	0.10	0.37	0.00	–0.01	0.01	0.01
mAb2^b^	0.019	0.03	0.44	0.11	0.41	0.016	0.03	0.45	0.10	0.41	0.00	0.01	–0.01	0.00
mAb3	0.033	0.02	0.50	0.09	0.37	0.034	0.03	0.48	0.09	0.39	0.01	–0.02	0.00	0.02
mAb4^b^	0.039	0.03	0.49	0.10	0.38	0.040	0.03	0.45	0.10	0.40	0.00	–0.04	0.00	0.02
mAb5	0.038	0.03	0.47	0.10	0.38	0.034	0.01	0.46	0.11	0.39	–0.02	–0.01	0.01	0.01
mAb6	0.023	0.03	0.46	0.10	0.40	0.027	0.03	0.45	0.10	0.40	0.00	–0.01	0.00	0.00
mAb7^a^	0.041	0.03	0.48	0.10	0.38	0.031	0.05	0.41	0.11	0.41	0.02	–0.07	0.01	0.03
mAb8^a^	0.027	0.03	0.44	0.10	0.41	0.027	0.06	0.39	0.12	0.42	0.03	–0.05	0.02	0.01
mAb9^a^	0.036	0.04	0.43	0.11	0.41	0.016	0.07	0.37	0.13	0.44	0.03	–0.06	0.02	0.03
mAb10	0.043	0.03	0.47	0.10	0.39	0.038	0.04	0.44	0.11	0.40	0.01	–0.03	0.01	0.01
mAb11^a^	0.036	0.03	0.50	0.09	0.37	0.035	0.05	0.41	0.11	0.42	0.02	–0.09	0.02	0.05
mAb12	0.039	0.04	0.44	0.11	0.39	0.028	0.06	0.42	0.11	0.42	0.02	–0.02	0.00	0.03
mAb13	0.035	0.04	0.46	0.10	0.38	0.027	0.03	0.45	0.10	0.40	–0.01	–0.01	0.00	0.02
mAb14	0.033	0.03	0.47	0.10	0.39	0.037	0.04	0.45	0.11	0.39	0.01	–0.02	0.01	0.00

a Samples with >5% change in secondary structure content.

b Samples in 50 mM sodium phosphate at pH 5.

### Influence of Acid-Induced Protein Denaturation on Near-UV CD Signal

Comparisons of near-UV spectra (Figure 2) of mAb samples at pH 3 (red traces) with those at higher pHs of 5 or 7 (blue traces) highlight conformational differences of varying magnitude and severity arising from changes to interactions of aromatic residues with one another as well as to their environment. These differences are not strictly correlated to variable backbone CD from far-UV spectra. To illustrate this point, mAbs 1 and 3 display little alteration in their far-UV spectra at pH 3 cf. pH 7, indicating no change in the backbone structure but exhibit large changes in the near-UV CD signal in the exciton “twin peak” region (namely increased and decreased intensities for the 230 and 240 nm peaks, respectively), indicating loss of π–π interactions. mAb5 is an interesting case. Its near-UV spectra at pH 3 and 7 are very similar (Figure 2), indicating little change in the π–π interactions. The corresponding far-UV spectra show a significant difference in magnitudes, though key features such as the positions of maxima and where it crosses the *x*-axis are similar (Figure 1). The secondary structure assignments for mAb5 at pH 3 and 7 were virtually identical (Table 2), though the pH 3 NRMSD is 50% larger than that at pH 7. We believe these differences can be attributed to loosening of the backbone structure (far-UV CD) without influencing the aromatic environment (near-UV CD).

## Conclusions

The secondary structure of antibodies has been analyzed extensively in the past by far-UV CD. However, comprehensive studies performed with large numbers of antibodies aiming to link far- and near-UV CD signals to antibody structural changes have not been performed until now. In this study, a carefully selected cohort of 14 purified therapeutically relevant mAbs, differing with respect to the IgG subclass and CDRs, has been examined by high-quality far- and near-UV SRCD in native (pH 5 or 7) and acid-stressed (pH 3) states. Deconvolution of SRCD far-UV spectra of the mAb cohort using currently popular algorithms and available reference sets revealed that it is the quality of the reference sets that exerts the greatest impact on deconvolution performance. For antibodies, SP175 and SMP180 can be recommended. Regardless of the analysis program, these two reference sets consistently assigned accurate secondary structures. Low spectral NRMSD values (often used as indicators of deconvolution quality) do not ensure accurate secondary structure determination and should be interpreted cautiously, ideally in combination with δ values determined from crystal structures or antibody homology models.

SRCD of the mAb cohort revealed significant spectral differences in the far-UV region (i.e., magnitude of the 201–202 nm peak, and absence/presence of a shoulder at around 190 nm) and even greater variations in near-UV CD (i.e., presence or absence of an exciton couplet signature between ∼230 and ∼250 nm, and peak intensity at 290 nm). The variety of spectra collected here for different therapeutic monoclonal antibodies under the same conditions illustrates CD’s utility as a biophysical tool for measuring subtle differences and gauging product quality. However, the complexity of signal changes makes the correct interpretation of data challenging, especially for nonexperts. Two spectral features identified in this study as particularly sensitive to changes in solution pH, namely, the positive 201–202 nm peak in the far-UV region and the negative exciton couplet in the near-UV region at around 230–240 nm, invite future investigation as single-wavelength reporters of mAb structure quality in CD-based PATs. Importantly, both wavelength ranges lie well within the technical capabilities of high-throughput and in-line capillary CD systems developed in our previous work.^29,30^ Finally, a strong recommendation to come out of this study is that CD measurements on antibodies and related molecules are best conducted across both regions of the UV spectrum, as each provides different and complementary information on their folding/mis-folding states.

## Supporting Information Available

Additional information on MOE models; limitations of benchtop CD instruments; challenges of mAb deconvolution; deconvolution results for reference data sets 2 and 5; quality and accuracy results for the deconvolution analysis; absorbance traces for SRCD data; DLS results and CD spectra comparison of SP175 against the panel of mAbs (PDF)
