# Supplementary material for: Analysis of the Structure of 14 Therapeutic Antibodies Using Circular Dichroism Spectroscopy
Source: Anal Chem. 2024 Sep 10;96(38):15151–9. doi: 10.1021/acs.analchem.4c01882 (PMC11428090; doi:10.1021/acs.analchem.4c01882)
Supplement: Supplementary file 1 — ac4c01882_si_001.pdf [file ac4c01882_si_001.pdf]

Supporting Information

for

Analysis of the Structure of 14 Therapeutic Antibodies Using Circular Dichroism Spectroscopy

Maria G. Bruque,[1,2] Alison Rodger,[3] Søren Vrønning Hoffmann,[4] Nykola C. Jones,[4] Jean Aucamp,[5] Tim R. Dafforn[2,†], Owen R. T. Thomas,*[,1,†]

[1]School of Chemical Engineering and [2]School of Biosciences, University of Birmingham, Edgbaston, B15 2TT, U.K.

[3]School of Natural Sciences, Macquarie University, NSW, 2109, Australia

[4]ISA, Department of Physics and Astronomy, Aarhus University, 8000 Aarhus C, Denmark

[5]Lonza Biologics, Slough, SL1 4DX, U.K.

†Joint contribution

*Corresponding author

Owen R.T. Thomas

Phone: +44 0 121 414 5278. E-mail: o.r.t.thomas@bham.ac.uk

Table of Contents

**Text S1.** Antibody homology models.	S-3
**Text S2.** Limitations of bench top instruments for measuring CD spectra of proteins.	S-4
**Text S3.** Challenges to the deconvolution of CD spectra of antibodies.	S-5
**Table S1.** Goodness-of-fit parameter NRMSD of analysis algorithms with reference data sets 2 and 5 for the mAb cohort.	S-6
**Table S2.** Average secondary structure content for the panel of mAbs determined by algorithms using reference data sets 2 and 5.	S-6
**Table S3.** Performance (based on δ values, eq. 2) of algorithms for determining secondary structure of the mAb cohort using reference data sets 2 and 5.	S-6
**Table S4.** Final templates used by MOE for loop grafting.	S-7
**Table S5.** Structure assessment of the mAb homology models for stereochemistry (MolProbity) and model quality estimation (QMEAN).	S-8
**Table S6.** Goodness-of-fit parameter NRMSD (eq. 1) of analysis algorithms with different reference sets for the mAb cohort.	S-9
**Table S7.** Average secondary structure content for the mAb cohort.	S-10
**Table S8**. Performance (based on δ values, eq. 2) of algorithms for determining secondary structure of the mAb cohort using different reference datasets.	S-11
**Figure S1.** Far-UV (A) and Near-UV (B) SRCD for the 14 mAbs (Table 1) in 50 mM sodium phosphate pH 5 for mAbs 2 and 4, or pH 7 for all other mAbs.	S-12
**Figure S2.** Absorbance curves corresponding to the Far-UV SRCD for the 14 mAbs (Table 1) in 50 mM sodium phosphate buffers. Panels: A – pH 5 for mAbs 2 and 4 and pH 7 for all other mAbs; B – pH 3 for all mAbs.	S-13
**Figure S3.** Absorbance curves corresponding to the Near-UV SRCD for the 14 mAbs (Table 1) in 50 mM sodium phosphate buffers. Panels: A – pH 5 for mAbs 2 and 4 and pH 7 for all other mAbs; B – pH 3 for all mAbs.	S-13
**Figure S4.** Mean radii for the 14 mAbs (Table 1) in 50 mM sodium phosphate pH 3, 5 and 7 determined by DLS.	S-14
**Figure S5.** Far-UV CD spectra of immunoglobulin G in SP175 dataset (PCDDB dataset CD0000039100, red) and the 14 mAb samples (black) at pH 7 (pH 5 for mAbs 2 and 4).	S-14

Text S1. Antibody homology models.

All proteins employed in this study share a common immunoglobulin (Ig) scaffold but differ with respect to IgG subclass and complementarity-determining regions (CDRs). The conserved Ig scaffold makes it possible to generate antibody structures by homology modeling without the need for high-resolution structural data (Sivasubramanian et al., 2009). Specialized modeling software for antibodies have thus emerged, and the full-length homology models they create has yielded excellent results with antibody sequences (Maier, 2014). In this study we have exploited such tools to generate antibody homology models for all 14 mAbs (Table 1) using the knowledge-based computer-aided molecular design platform “Molecular Operating Environment, MOE” (Chemical Computing Group 2022). MOE accesses a database of antibody structures available in the Protein Data Bank (PDB). Full-length light and heavy chain sequences were submitted to the antibody modeler application with “Ig” as model type and refinement set to “1”. Selection of suitable framework templates for VL and VH was done automatically based on sequence identity, sequence similarity, global structure factors, and backbone assessment. The templates for the three CDR loops in the light (L1, L2, L3) and heavy (H1, H2 H3) chains were also assigned automatically based on sequence similarity and length, and representative conformations. The fragment crystallizable (Fc) region templates were chosen from the MOE antibody library on the basis of mAb isotype: 1HZH (Saphire et al., 2001) for IgG1; 4HAF (Teplyakov et al., 2013) for IgG2; and 5DK3 (Scapin et al., 2015) for IgG4. All selected templates had average identity scores of 100% (or 95.6% for mAb-7 VL) ensuring that they are ideal protein structures for homology modeling. Loop grafting was then used to combine the chosen templates (Table S4) and create the complete full-length antibody model. The models were optimized to reduce Ramachandran and rotamer outliers as well as any atom clashes. Final models were further optimized by applying a global energy minimization with a gradient of 0.1 RMS (root-mean-square). Model evaluation was performed on the final models using MolProbity and QMEANZ score values which inform on the quality of the stereochemical and overall model *cf.* to that of experimental structures (Table S5; Chen et al., 2010; Benekrt et al., 2011).

REFERENCES

Sivasubramanian, A.; Sircar, A.; Chaudhury, S.; Gray, J.J. Toward high-resolution homology modeling of antibody Fv regions and application to antibody–antigen docking. *Proteins* **2009.** *74*, 497.

Maier, J.K.X.; Labute, P. Assessment of fully automated antibody homology modeling protocols in molecular operating environment. *Proteins Struct. Funct. Bioinf.* **2014.** *82*, 1599–1610.

Molecular Operating Environment (MOE), 2022.02 Chemical Computing Group ULC, 910-1010 Sherbrooke St. W., Montreal, QC H3A 2R7, Canada, 2024.

Saphire, E.O.; Parren, P.W.H.I.; Pantophlet, R.; Zwick, M.B.; Morris, G.M.; Rudd, P.M.; Dwek, R.A.; Stanfield, R.L.; Burton, D.R.; Wilson, I. A Crystal structure of a neutralizing human IGG against HIV-1: a template for vaccine design. *Science* **2001.** *293*, 1155–1159.

Teplyakov, A.; Zhao, Y.; Malia, T.J.; Obmolova, G.; Gilliland, G.L. IgG2 Fc structure and the dynamic features of the IgG CH2–CH3 interface. *Mol. Immunol*. **2013.** *56*, 131–139.

Scapin, G.; Yang, X.; Prosise, W.W.; McCoy, M.; Reichert, P.; Johnston, J.M.; Kashi, R.S.; Strickland, C. Structure of full-length human anti-PD1 therapeutic IgG4 antibody pembrolizumab. *Nat. Struct. Mol. Biol.* **2015.** *22*, 953–958.

Chen, V.B.; Arendall, W.B.; Headd, J.J.; Keedy, D.A.; Immormino, R.M.; Kapral, G.J.; Murray, L.W.; Richardson, J.S.; Richardson, D.C. MolProbity: all-atom structure validation for macromolecular crystallography. *Acta Crystallogr. Sect. D. Biol. Crystallogr.* **2010.** *66*, 12–21.

Benkert, P.; Biasini, M.; Schwede, T. Toward the estimation of the absolute quality of individual protein structure models. *Bioinformatics* **2011.** *27*, 343–350.

Text S2. Limitations of bench top instruments for measuring CD spectra of proteins.

Generally, measurement of CD spectra of proteins involves collecting data from two distinct regions of the electromagnetic spectrum, i.e., near-UV and far-UV. Near-UV (230-330 nm) CD provides data on electronic transitions of the aromatic side chains of Tyr, Trp and Phe residues, and of disulfide bonds (Kelly et al., 2005; Nordén et al., 2010; Berova et al., 2000). The former contributes information on the arrangement of aromatic residues in a given protein and is therefore useful for probing changes to its hydrophobic core. Near-UV CD can be measured by most laboratory-based instruments but is of low intensity; key demands therefore are the use of long pathlengths and/or high protein concentrations (Burns, 1994). Far-UV (<240 nm) CD probes the electronic transitions in the peptide bonds, providing information on secondary structural rearrangements in the protein. A major attraction of far-UV over near-UV is that far- UV spectra can be quantitatively deconvolved to produce estimates of secondary structure content (Berova et al., 2000). However, far-UV spectra are more challenging to collect (*cf.* near-UV) because buffer components, salts and even water absorb light at short wavelengths (Greenfield, 2007); sodium phosphate buffers were selected for their relative transparency in the far-UV *cf.* other commonly employed buffers. Several important electronic features occur below 200 nm (Wallace and Janes, 2001), but these are poorly accessed by most "bench top" instruments, which regardless of sample, often struggle to collect data of sufficient quality much below 200 nm. This shortcoming is neatly addressed by SRCD.

REFERENCES

Kelly, S.; Jess, T.; Price, N. How to study proteins by circular dichroism. Biochim. Biophys. Acta - Proteins Proteom. **2005.** *1751*, 119–139.

Nordén, B.; Rodger, A.; Dafforn, T. R. Linear Dichroism and Circular Dichroism: A Textbook on Polarized Spectroscopy; The Royal Society of Chemistry: Cambridge, 2010.

Berova, N.; Nakanishi, K.; Woody, R. Circular dichroism principles and applications; W. Wiley-VCH: New York, 2000.

Burns, D. Analytical Applications of Circular Dichroism. *Anal. Chim. Acta* **1994.** *292*, 215.

Greenfield, N. J. Using circular dichroism spectra to estimate protein secondary structure. *Nat. Protoc.* **2007.** *1*, 2876–2890.

Wallace, B. A.; Janes, R. W. Synchrotron radiation circular dichroism spectroscopy of proteins: secondary structure, fold recognition and structural genomics. *Curr. Opin. Chem. Biol.* **2001.** *5*, 567–571.

Text S3. Challenges to the deconvolution of CD spectra of antibodies.

Far-UV CD has been shown to contain quantitative information on the secondary structural elements present in proteins. Several deconvolution algorithms have been developed that provide good estimates of the proportions of each type of secondary structure within a protein from the far-UV CD spectrum (Hall et al., 2013). Most deconvolution algorithms applied to CD data analysis use reference datasets containing CD spectra of proteins with known secondary structure. The reference datasets employed for deconvolution generally depend on the protein being analyzed. To simplify data analysis, resources such as DichroWeb (Whitmore and Wallace, 2004) have a library of reference datasets which contain both different reference sets and different fitting algorithms. The large structural and spectral diversity of β-sheets makes the deconvolution of CD spectra for proteins with high β-sheet content (e.g. antibodies) especially challenging given that they are, at least in part, poorly represented in reference sets. The BeStSel (Beta Structure Selection) method was specifically developed and optimized to analyze the secondary structure content of β-sheet rich proteins to address this problem (Micsonai et al., 2015; Micsonai et al., 2018).

REFERENCES

Hall, V.; Nash, A.; Hines, E.; Rodger, A. Elucidating protein secondary structure with circular dichroism and a neural network. *J. Comput. Chem.* **2013.** *34*, 2774–2786.

Whitmore, L.; Wallace, B. A. DICHROWEB, an online server for protein secondary structure analyses from circular dichroism spectroscopic data. *Nucleic Acids Res.* **2004.** *32*, W668.

Micsonai, A.; Wien, F.; Kernya, L.; Lee, Y. H.; Goto, Y.; Réfrégiers, M.; Kardos, J. Accurate secondary structure prediction and fold recognition for circular dichroism spectroscopy. *Proc. Natl. Acad. Sci. USA* **2015.** *112*, E3095–E3103.

Micsonai, A.; Wien, F.; Bulyáki, É.; Kun, J.; Moussong, É.; Lee, Y. H.; Goto, Y.; Réfrégiers, M.; Kardos, J. BeStSel: a web serv-er for accurate protein secondary structure prediction and fold recognition from the circular dichroism spectra. *Nucleic Acids Res.* **2018.** *46*, W315–W322.

Table S1. Goodness-of-fit parameter NRMSD of analysis algorithms with reference data sets 2 and 5 for the mAb cohort.

Program	Ref.	mAb1	mAb2	mAb3	mAb4	mAb5	mAb6	mAb7	mAb8	mAb9	mAb10	mAb11	mAb12	mAb13	mAb14
CONTINLL	Set 2	0.219	0.19	0.196	0.203	0.176	0.168	0.203	0.187	0.198	0.193	0.212	0.164	0.178	0.16
	Set 5	0.224	0.182	0.206	0.215	0.172	0.182	0.211	0.181	0.203	0.211	0.222	0.159	0.195	0.175
SELCON3	Set 2	0.234	0.511	0.273	0.258	0.249	N/A	0.379	N/A	0.441	N/A	0.261	0.183	0.427	0.32
	Set 5	N/A	N/A	N/A	N/A	N/A	0.631	N/A	0.667	N/A	0.354	N/A	0.356	N/A	N/A
CDSSTR	Set 2	0.059	0.017	0.031	0.039	0.04	0.026	0.04	0.025	0.028	0.034	0.042	0.045	0.047	0.038
	Set 5	0.051	0.027	0.042	0.049	0.043	0.039	0.059	0.039	N/A	0.055	0.056	0.062	0.055	0.048

Table S2. Average secondary structure content for the panel of mAbs determined by algorithms using reference data sets 2 and 5.

Program	Ref.	Helix*	Sheets*	Turns*	Unordered*
CONTINLL	Set 2	0.09	0.34	0.14	0.43
	Set 5	0.11	0.39	0.27	0.23
SELCON3	Set 2	0.09	0.35	0.11	0.46
	Set 5	0.32	0.36	0.24	0.18
CDSSTR	Set 2	0.05	0.36	0.16	0.41
	Set 5	0.07	0.35	0.31	0.27

*The secondary structure assignments used were: regular alpha helix (helix 1, αR), distorted alpha helix (helix 2, αD), 3_{10}-helix (3/10) and Polyproline II helix (P2) were grouped and classified as “Helix”, regular beta sheet (sheet 1, βR) and distorted beta sheet (sheet 2, βD) were assigned to “Sheets”, turns (T) were classified as “Turns” and unordered (U) structures as “Unordered”.

Table S3. Performance (based on δ values, eq. 2) of algorithms for determining secondary structure of the mAb cohort using reference data sets 2 and 5.

Program	Ref.	Helix	Sheets	Turns	Unordered
CONTINLL	Set 2	0.02	0.14	0.03	0.10
	Set 5	0.05	0.09	0.15	0.11
SELCON3	Set 2	0.02	0.11	0.02	0.11
	Set 5	0.15	0.08	0.08	0.09
CDSSTR	Set 2	0.02	0.12	0.05	0.08
	Set 5	0.02	0.13	0.19	0.07

Table S4. Final templates used by MOE for loop grafting.

Sample	Framework		CDR Loop					
	VL	VH	L1	L2	L3	H1	H2	H3
mAb1	4YWG.M	4YWG.I	4YWG.M	4YWG.M	4YWG.M	4YWG.I	4YWG.I	4YWG.I
mAb2	1IGM.L	1IGM.H	1IGM.L	1IGM.L	1IGM.L	1IGM.H	1IGM.H	1IGM.H
mAb3	4RIS.L	4RIS.H	4RIS.L	4RIS.L	4RIS.L	4RIS.H	4RIS.H	4RIS.H
mAb4	4D9Q.D	4D9Q.E	4D9Q.D	4D9Q.D	4D9Q.D	4D9Q.E	4D9Q.E	4D9Q.E
mAb5	6XMI.A	6XMI.B	7MN8.C	7MN8.C	7MN8.C	7MN8.D	7MN8.D	7MN8.D
mAb6	6ML8.L	6ML8.H	6ML8.L	6ML8.L	6ML8.L	6ML8.H	6ML8.H	6ML8.H
mAb7	4YDV.L	4YDV.H	5EA0.L	4YDV.L	4YDV.L	AYDV.H	4YDV.H	4YDV.H
mAb8	4DQO.L	4DQO.H	MME.L	4DQO.L	4DQO.L	4DQO.H	4DQO.H	4DQO.H
mAb9	1BBJ.A	1BBJ.B	1BBJ.A	1BBJ.A	1BBJ.A	1BBJ.B	1BBJ.B	1BBJ.B
mAb10	5SX5.K	5SX5.J	5SX5.K	5SX5.K	5SX5.K	5SX5.J	5SX5.J	5SX5.J
mAb11	3ULU.C	3ULU.C	3ULU.C	3ULU.C	3ULU.C	3ULU.C	3ULU.C	3ULU.C
mAb12	4OSU.L	4OSU.H	4OSU.L	4OSU.L	4OSU.L	4OSU.H	4OSU.H	4OSU.H
mAb13	4NM4.L	4NM4.H	4NM4.L	4NM4.L	4NM4.L	4NM4.H	4NM4.H	4NM4.H
mAb14	4KAQ.L	4KAQ.H	4KAQ.L	4KAQ.L	4KAQ.L	4KAQ.H	4KAQ.H	4KAQ.H

Table S5. Structure assessment of the mAb homology models for stereochemistry (MolProbity) and model quality estimation (QMEAN).

Sample	MolProbity										QMEANDisCo					
	MolProbity score	Clash score	Ramachandran		Rotamer outliers	C-Beta deviations	Bad bonds	Bad angles	Cis prolines	Twisted non-proline	Global	QMEAN	CB	All atom	Solvation	Torsion
			Favoured	Outliers												
mAb1	1.74	0.49	90.78	0.9	5.26	13	4/10642	75/14514	13/96	14/1242	0.74	-1.07	-0.41	0.06	-2.63	-0.16
mAb2	1.78	0.54	90.47	1.21	5.38	9	4/10504	51/14304	12/90	10/1236	0.74	-1.54	0.15	0.25	-2.52	-0.74
mAb3	1.6	0.25	90.55	1.5	4.22	12	5/10628	57/14492	8/100	14/1238	0.72	-1.51	-0.22	0.12	-2.67	-0.6
mAb4	1.58	0.3	91.45	0.76	4.02	10	4/10398	46/14168	13/92	16/1222	0.76	-0.54	0.08	0.48	-2.18	0.13
mAb5	1.59	0.74	91.36	1.21	2.83	10	5/10530	46/14344	13/92	14/1232	0.76	-1	0.37	0.39	-2.69	-0.2
mAb6	1.62	0.29	90.34	1.55	4.18	9	5/10736	48/14604	13/88	11/1272	0.74	-1.57	-0.32	0.28	-2.78	-0.62
mAb7	1.63	0.43	91.89	0.82	4.36	9	4/10790	45/14692	13/94	12/1254	0.74	-1.28	-0.38	0.18	-2.47	-0.42
mAb8	1.62	0.34	90.19	1.25	3.94	11	4/10756	59/14646	8/96	15/1264	0.71	-1.58	-1.1	0.12	-2.34	-0.64
mAb9	1.78	0.4	89.25	1.38	5.6	11	4/10406	59/14154	13/82	22/1224	0.74	-1.93	0.29	0.43	-2.47	-1.17
mAb10	1.7	0.4	88.85	1.6	4.24	9	4/10436	81/14224	12/92	19/1222	0.74	-1.63	0.21	0.5	-2.96	-0.71
mAb11	1.77	0.9	88.51	2.36	3.42	21	4/10458	69/14286	11/106	19/1212	0.71	-1.87	-0.02	0.37	-2.3	-1.12
mAb12	1.59	0.25	91.51	0.75	4.44	12	4/10534	56/14360	9/102	12/1244	0.73	-1.45	-0.8	0.05	-2.42	-0.53
mAb13	1.72	0.54	90.63	1.12	4.57	14	4/10666	50/14528	13/88	19/1250	0.75	-1.37	-0.31	0.3	-2.2	-0.61
mAb14	1.58	0.25	90.76	1.52	3.96	6	4/10480	48/14282	13/96	15/1228	0.74	-1.68	-0.6	0.39	-2.53	-0.76

Table S6. Goodness-of-fit parameter NRMSD (eq. 1) of analysis algorithms with different reference sets for the mAb cohort.

Analysis	Ref. set	mAb1	mAb2	mAb3	mAb4	mAb5	mAb6	mAb7	mAb8	mAb9	mAb10	mAb11	mAb12	mAb13	mAb14
	Set 1	0.203	0.164	0.211	0.226	0.194	0.198	0.216	0.182	0.222	0.192	0.216	0.163	0.202	0.19
	Set 3	0.203	0.128	0.225	0.212	0.199	0.17	0.208	0.17	0.167	0.174	0.207	0.156	0.205	0.204
	Set 4	0.211	0.173	0.229	0.192	0.171	0.164	0.21	0.189	0.112	0.255	0.19	0.119	0.192	0.214
	Set 6	0.203	0.128	0.225	0.212	0.199	0.17	0.208	0.17	0.167	0.174	0.207	0.156	0.205	0.204
CONTIN	Set 7	0.211	0.173	0.229	0.192	0.171	0.164	0.21	0.189	0.112	0.255	0.19	0.119	0.192	0.214
	SP175	0.137	0.041	0.092	0.151	0.099	0.099	0.101	0.082	0.178	0.093	0.174	0.206	0.141	0.078
	SP175t	0.145	0.142	0.125	0.173	0.146	0.101	0.162	0.149	0.109	0.112	0.142	0.133	0.144	0.105
	SMP180	N/A	0.046	0.099	0.159	N/A	0.108	0.327	N/A	0.198	N/A	0.393	N/A	0.139	0.078
	SMP180t	0.147	0.158	0.157	0.106	0.209	0.105	0.097	0.113	0.113	0.251	0.159	0.098	0.107	0.106
	Set 1	0.297	N/A	0.353	0.793	0.229	0.472	0.3	0.467	0.441	0.198	0.348	0.302	N/A	0.695
	Set 3	0.453	0.383	0.474	0.419	0.32	0.315	0.373	0.332	0.174	0.302	0.629	0.312	0.284	0.42
	Set 4	0.277	0.256	0.476	0.297	0.203	0.138	0.27	0.206	0.315	0.248	0.22	0.176	0.34	0.31
SELCON3	Set 6	0.273	0.357	0.25	0.229	0.179	0.25	0.155	0.244	0.238	0.301	0.367	0.24	0.233	0.2
	Set 7	0.319	0.256	0.399	0.301	0.264	0.145	0.27	0.183	0.31	0.26	0.207	0.246	0.328	0.278
	SP175t	0.472	0.566	0.449	0.553	0.564	0.432	0.297	0.504	1.063	0.326	0.382	0.386	0.383	0.347
	SMP180	0.556	0.848	0.568	0.642	0.712	0.777	0.658	0.775	0.787	0.852	0.57	N/A	0.740	0.675
	Set 1	0.056	0.025	0.039	0.048	0.039	0.033	0.048	0.033	0.033	0.052	0.041	0.042	0.059	0.043
	Set 3	0.053	0.037	0.046	0.08	0.069	0.055	0.067	0.064	0.063	0.048	0.055	0.068	0.066	0.061
	Set 4	0.043	0.029	0.034	0.055	0.056	0.051	0.056	0.052	0.054	0.045	0.039	0.056	0.043	0.052
	Set 6	0.051	0.034	0.047	0.072	0.069	0.054	0.067	0.06	0.062	0.043	0.054	0.071	0.058	0.056
CDSSTR	Set 7	0.038	0.028	0.035	0.053	0.054	0.048	0.052	0.049	0.053	0.04	0.038	0.057	0.049	0.057
	SP175	0.041	0.019	0.033	0.039	0.038	0.023	0.041	0.027	0.036	0.043	0.036	0.039	0.035	0.033
	SP175t	0.035	0.03	0.034	0.053	0.034	0.03	0.04	0.042	0.038	0.032	0.031	0.031	0.038	0.031
	SMP180	0.066	0.03	0.055	0.066	0.048	0.051	0.06	0.047	0.058	0.054	0.063	0.05	0.073	0.057
	SMP180t	0.066	0.03	0.04	0.061	0.041	0.034	0.046	0.047	0.051	0.036	0.044	0.053	0.042	0.041
BeStSel		0.021	0.022	0.020	0.024	0.020	0.024	0.020	0.019	0.028	0.016	0.022	0.014	0.019	0.017

Table S7. Average secondary structure content for the mAb cohort.

Program	Reference Set	H	S	T	U
CONTINLL	Set 1	0.01	0.42	0.25	0.32
	Set 3	0.03	0.41	0.21	0.35
	Set 4	0.04	0.41	0.22	0.34
	Set 6	0.06	0.47	0.28	0.19
	Set 7	0.04	0.42	0.24	0.30
	SP175	0.05	0.47	0.10	0.37
	SP175t	0.06	0.44	0.11	0.38
	SMP180	0.05	0.47	0.11	0.37
	SMP180t	0.04	0.48	0.11	0.38
SELCON3	Set 1	0.02	0.40	0.23	0.35
	Set 3	0.01	0.43	0.22	0.36
	Set 4	0.02	0.43	0.21	0.33
	Set 6	0.02	0.47	0.24	0.27
	Set 7	0.02	0.43	0.21	0.33
	SP175t	0.06	0.46	0.11	0.37
	SMP180	-0.01	0.49	0.10	0.38
CDSSTR	Set 1	-0.01	0.34	0.26	0.39
	Set 3	0.00	0.41	0.23	0.34
	Set 4	0.03	0.41	0.24	0.32
	Set 6	0.01	0.41	0.24	0.33
	Set 7	0.01	0.41	0.24	0.34
	SP175	0.03	0.47	0.10	0.39
	SP175t	0.02	0.46	0.11	0.39
	SMP180	0.03	0.46	0.11	0.39
	SMP180t	0.01	0.51	0.10	0.36
BeStSel		0.00	0.45	0.07	0.48
DSSP of homology models		0.07	0.48	0.12	0.33
DSSP of IgG crystal structures		0.07	0.44	0.10	0.39

Table S8. Performance (based on δ values, eq. 2) of algorithms for determining secondary structure of the mAb cohort using different reference datasets.

Program	Reference Set	H	S	T	U
CONTINLL	Set 1	0.06	0.06	0.14	0.02
	Set 3	0.04	0.07	0.09	0.03
	Set 4	0.04	0.07	0.11	0.02
	Set 6	0.01	0.03	0.16	0.15
	Set 7	0.03	0.06	0.12	0.04
	SP175	0.02	0.02	0.02	0.04
	SP175t	0.01	0.04	0.01	0.05
	SMP180	0.02	0.02	0.01	0.03
	SMP180t	0.04	0.02	0.01	0.05
SELCON3	Set 1	0.05	0.07	0.11	0.03
	Set 3	0.07	0.06	0.10	0.03
	Set 4	0.05	0.06	0.10	0.03
	Set 6	0.06	0.03	0.13	0.08
	Set 7	0.06	0.07	0.10	0.02
	SP175t	0.02	0.03	0.02	0.04
	SMP180	0.08	0.03	0.02	0.05
CDSSTR	Set 1	0.08	0.14	0.15	0.07
	Set 3	0.07	0.07	0.12	0.02
	Set 4	0.04	0.08	0.12	0.02
	Set 6	0.07	0.07	0.12	0.02
	Set 7	0.07	0.07	0.12	0.01
	SP175	0.04	0.03	0.02	0.06
	SP175t	0.05	0.03	0.02	0.06
	SMP180	0.04	0.03	0.01	0.06
	SMP180t	0.06	0.04	0.02	0.03
BeStSel		0.07	0.04	0.05	0.15

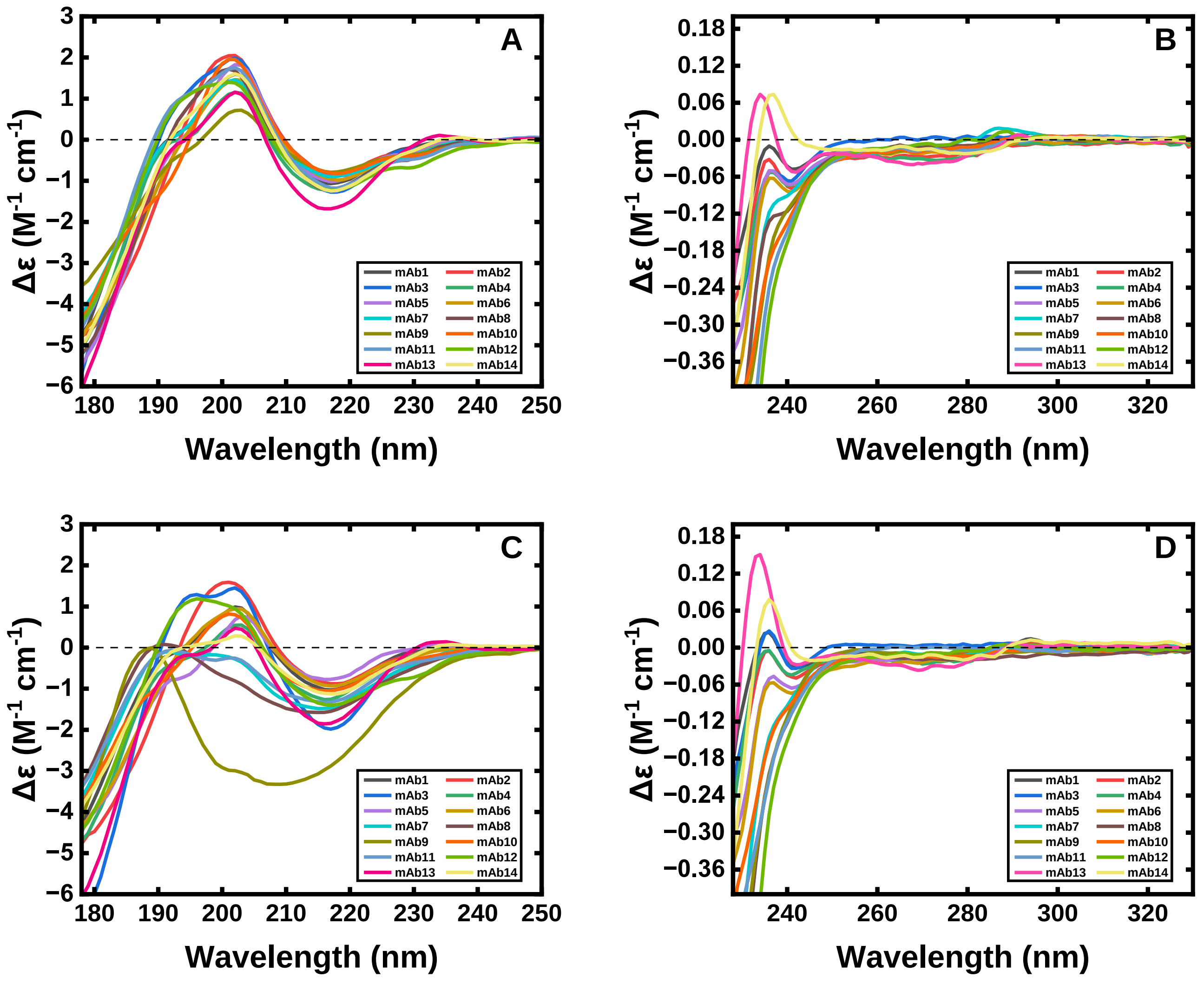

Figure S1. Far-UV (A) and Near-UV (B) SRCD for the 14 mAbs (Table 1) in 50 mM sodium phosphate pH 5 for mAbs 2 and 4, or pH 7 for all other mAbs.

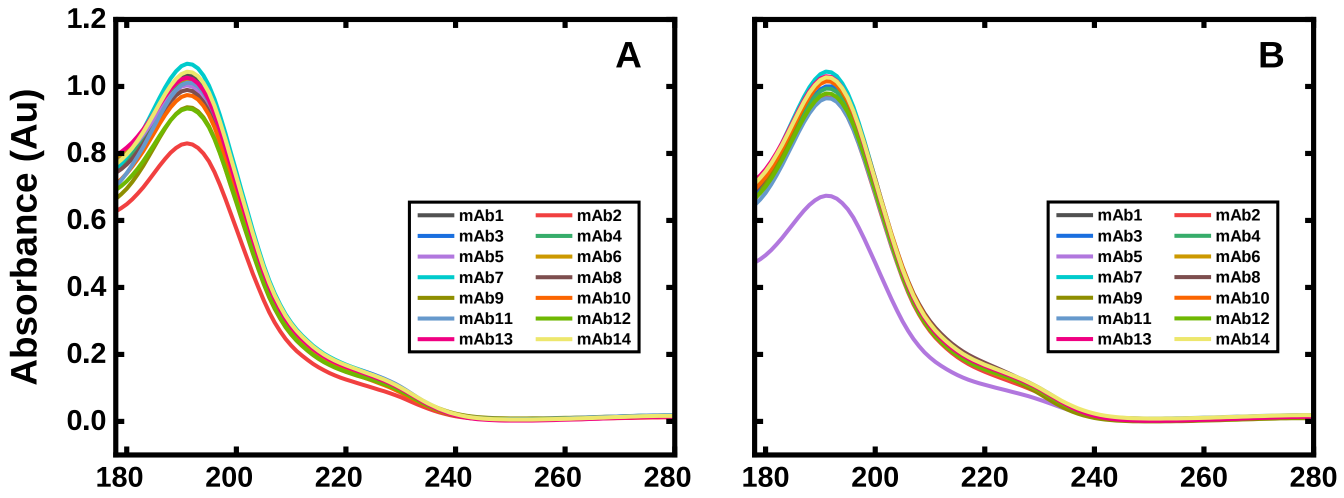

Figure S2. Absorbance curves corresponding to the Far-UV SRCD for the 14 mAbs (Table 1) in 50 mM sodium phosphate buffers. Panels: A – pH 5 for mAbs 2 and 4 and pH 7 for all other mAbs; B – pH 3 for all mAbs.

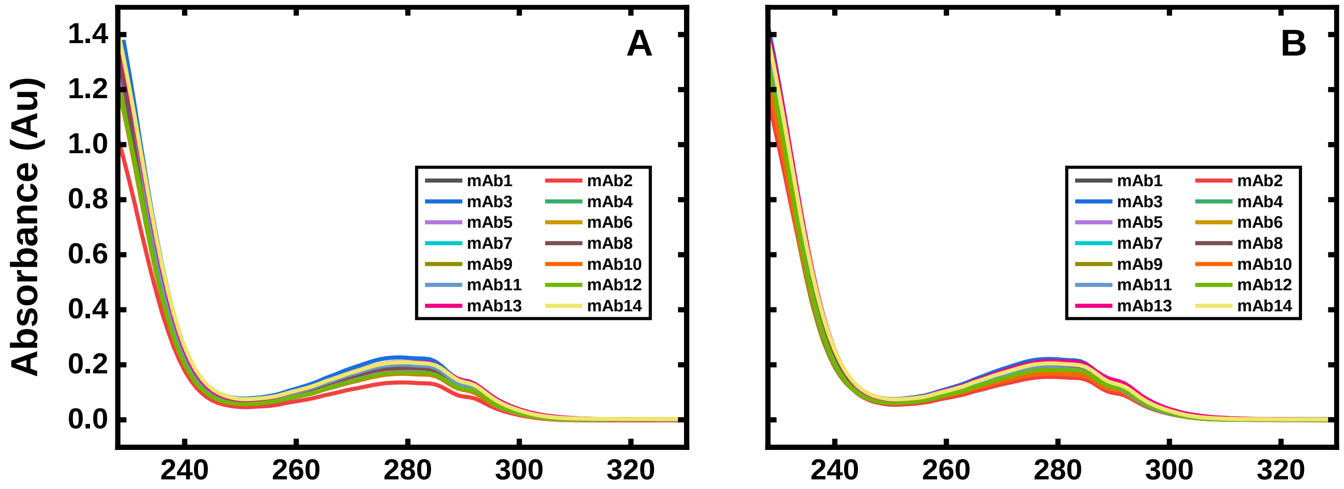

Figure S3. Absorbance curves corresponding to the Near-UV SRCD for the 14 mAbs (Table 1) in 50 mM sodium phosphate buffers. Panels: A – pH 5 for mAbs 2 and 4 and pH 7 for all other mAbs; B – pH 3 for all mAbs.

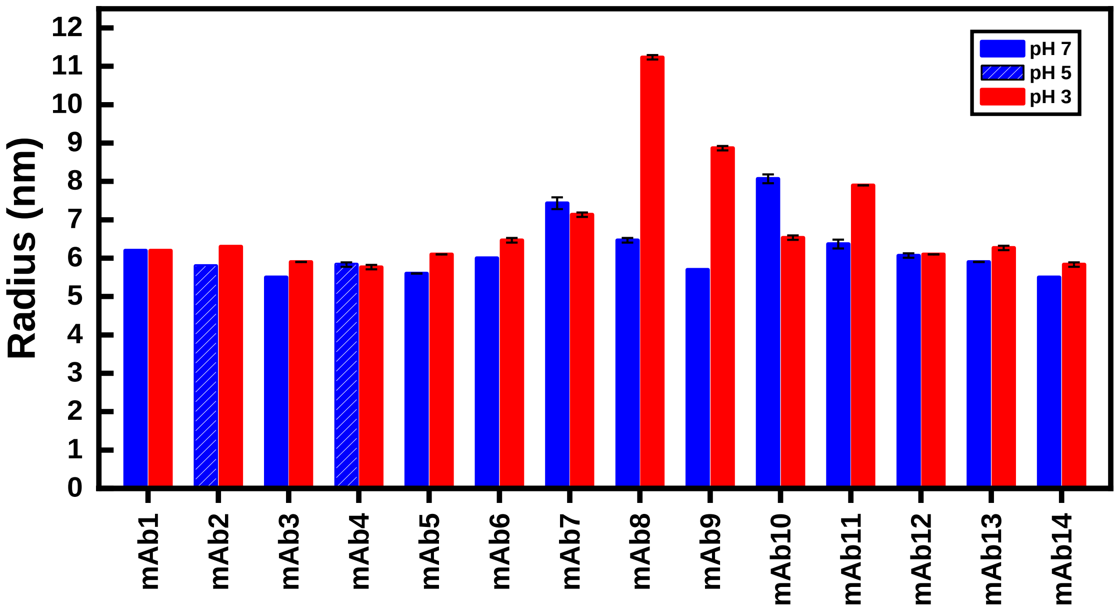

Figure S4. Mean radii for the 14 mAbs (Table 1) in 50 mM sodium phosphate pH 3, 5 and 7 determined by DLS.

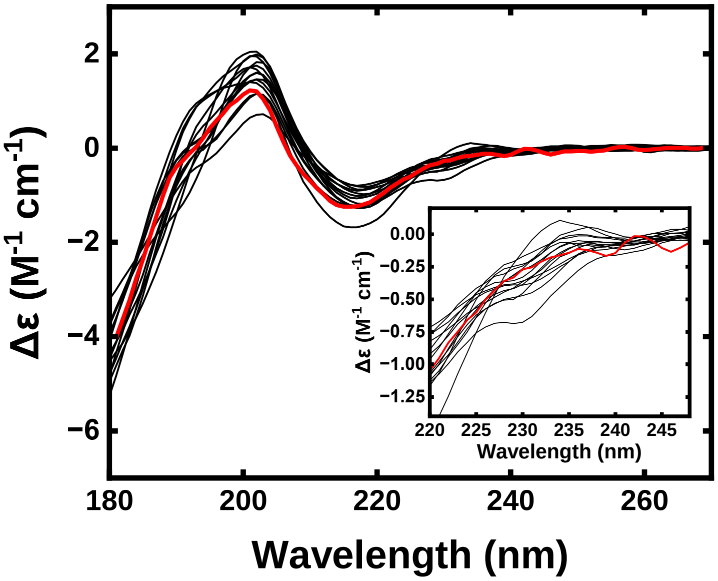

Figure S5. Far-UV CD spectra of immunoglobulin G in SP175 dataset (PCDDB dataset CD0000039100, red) and the 14 mAb samples (black) at pH 7 (pH 5 for mAbs 2 and 4).
